# Prevalence and characterization of post-acute sequelae of SARS-CoV-2 infection (PASC) in Rwanda

**DOI:** 10.1016/j.ijregi.2025.100738

**Published:** 2025-09-24

**Authors:** Fernand Rwamwejo, Vivianne Umuhire Niyonkuru, Gilbert Rukundo, Eric Remera, Edson Rwagasore, Les Sztandera, Charles Ruranga, Elizabeth Krebs

**Affiliations:** 1 Ministry of Health, Kigali, Rwanda; 2 Rwanda Biomedical Centre, Kigali, Rwanda; 3 Thomas Jefferson University, Philadelphia, USA; 4 University of Rwanda, Kigali, Rwanda

**Keywords:** Post-acute sequelae of SARS-CoV-2 infection (PASC), Long COVID, Prevalence, Risk Factors, Cross-sectional survey, Rwanda

## Abstract

**Objectives:**

Reliable, population-level estimates of post-acute sequelae of SARS-CoV-2 infection (PASC) remain scarce for sub-Saharan Africa. We aimed to quantify PASC prevalence and identify associated factors among adult COVID-19 survivors in Rwanda.

**Methods:**

A nationally representative cross-sectional telephone survey (August-October 2024) sampled 3143 adults from the national COVID-19 registry. PASC was defined as new or persisting symptoms ≥3 months after acute illness and lasting ≥2 months. The prevalence was calculated, and multivariable logistic regression identified factors independently associated with PASC.

**Results:**

Overall, PASC prevalence was 34%. Leading symptoms were back pain, headache, dizziness, fatigue, and reduced sexual desire. Higher odds of PASC occurred in women, adults ≥35 years, individuals with ≥2 COVID-19 infections, and those screening positive for anxiety. Current alcohol use was linked to lower odds. COVID-19 vaccination showed no association with PASC.

**Conclusions:**

Approximately one-third of adult Rwandan COVID-19 survivors continue to experience persistent symptoms. This burden signals that post-COVID care must become an integral part of routine health services, especially as new variants periodically drive fresh waves of infection. Preventing repeat infections and integrating mental health support emerge as actionable priorities. Harmonized longitudinal studies are needed to clarify PASC causality.

## Highlights

• Long COVID affected 34% of Rwandans, on average, 3 years after infection.
• Back pain, headache, dizziness, fatigue, and brain fog were the most common symptoms.
• Female sex, age ≥35 years, repeat infection, and anxiety predicted long COVID risk.
• Nationwide survey underscores need to integrate post-outbreak care into routine care.

## Introduction

Persistent symptoms after acute SARS-CoV-2 infection, widely referred to as long COVID, post-COVID condition, or post-acute sequelae of COVID-19 (PASC; term used hereafter), were first reported by patients in early 2020 [1]. To standardize research and clinical practice, the World Health Organization (WHO) convened a global Delphi panel in October 2021 that defined PASC as new, relapsing, or persistent symptoms emerging ≥3 months after confirmed or probable COVID-19 and lasting ≥2 months without an alternative explanation [2]. Adoption of this consensus definition has unified data collection and catalyzed large international consortia, resulting in hundreds of PASC studies worldwide.

Although African countries represent about 18% of the global population [3], published research remains limited compared with other regions. A 2025 update to the original 2022 systematic review and meta-analysis of the Global burden of PASC by Chen et al. [4] includes studies from Africa (nine studies), Asia (126 studies), Europe (195 studies), North America (61 studies), Oceania (three studies), and South America (31 studies)[5]. This updated meta-analysis is still under review for publication, but highlights the large contributions to the literature on PASC achieved by other regions while Africa remains minimally represented in proportion to the population it includes.

Rwanda’s rigorous nationwide testing and the Rwanda Biomedical Centre’s comprehensive registry provide one of the continent’s most complete national COVID-19 datasets, a valuable resource for developing infection control models and supporting other African countries with limited data. Rwanda has implemented a dedicated program focused on PASC care. This program is integrated as part of the existing non-communicable disease program and clinics in the country. It aims to identify individuals with PASC, train health care providers on PASC care, and enhance the management and support provided to those experiencing prolonged symptoms and complications after COVID-19. By integrating PASC care into the established health care system, Rwanda seeks to improve the diagnosis, treatment, and overall outcomes for individuals affected by this condition. Therefore, to inform the ongoing efforts in PASC care in Rwanda, we undertook a nationally representative analysis to estimate PASC prevalence, further understand its physical and psychosocial manifestations, and identify demographic, clinical, and epidemiological risk factors. These data will contribute to the development of targeted interventions, improved diagnostics, and more effective treatment strategies for Rwandans experiencing PASC while adding to the global body of knowledge, where too few Africans are represented currently.

## Methods

### Study design

This was a cross-sectional study, drawing a nationally representative sample from Rwanda’s COVID-19 registry to identify and characterize individuals with PASC.

### Setting

Rwanda is a geographically compact, lower-middle-income country in East Africa with an estimated population of 13.2 million [6]. The national health system is decentralized, comprising national referral hospitals, provincial and district hospitals, health centers, and a large network of community health workers. This network ensures service delivery across both urban and rural areas.

The Rwanda Biomedical Centre (RBC), the Ministry of Health’s national implementation agency, leads the country’s COVID-19 response, including surveillance, mass testing, case management, vaccination, and integration of PASC care into existing non-communicable disease clinics. Between March 2020 and March 2024, Rwanda conducted over 6 million COVID-19 tests with an overall positivity rate of 2.2%, supported by contact tracing, targeted community checkpoints, and outbreak containment measures.

The country’s integrated digital health and surveillance infrastructure, including the national COVID-19 registry, captures demographic and laboratory data from all provinces. This system provides a comprehensive national sampling frame, enabling inclusion of individuals across diverse socio-economic groups, geographic settings, and levels of COVID-19 severity.

For this study, trained staff used the national COVID-19 registry to identify eligible adults and conducted structured telephone interviews from August 8 to October 10, 2024.

### Participants

All positive test records in the RBC registry between March 14, 2020 and March 31, 2024 (n = 278,406) were initially screened for inclusion. First, records with multiple positive results for the same individual within 14 days were merged, retaining only the first positive test, which brought the total down to 133,406 records. Next, we filtered for individuals who were aged 18 years or older and were tested either because they had symptoms of COVID-19 or were suspected cases, defined as having had contact with a confirmed positive case. From these, we further narrowed down to those with a valid phone number (n = 127,976), and for individuals with multiple records, only the most recent record was retained, leaving us with 101,976 unique individuals.

Inclusion criteria:

• 18 years of age or older
• have tested positive for COVID-19 using reverse transcription-polymerase chain reaction or antigen tests since March 2020
• have their COVID-19 test recorded within the RBC COVID-19 database
• at least 3 months post their most recent positive COVID-19 test
• willing and able to provide informed consent.

Exclusion criteria:

• COVID-19 self-test results

**Figure 1:**
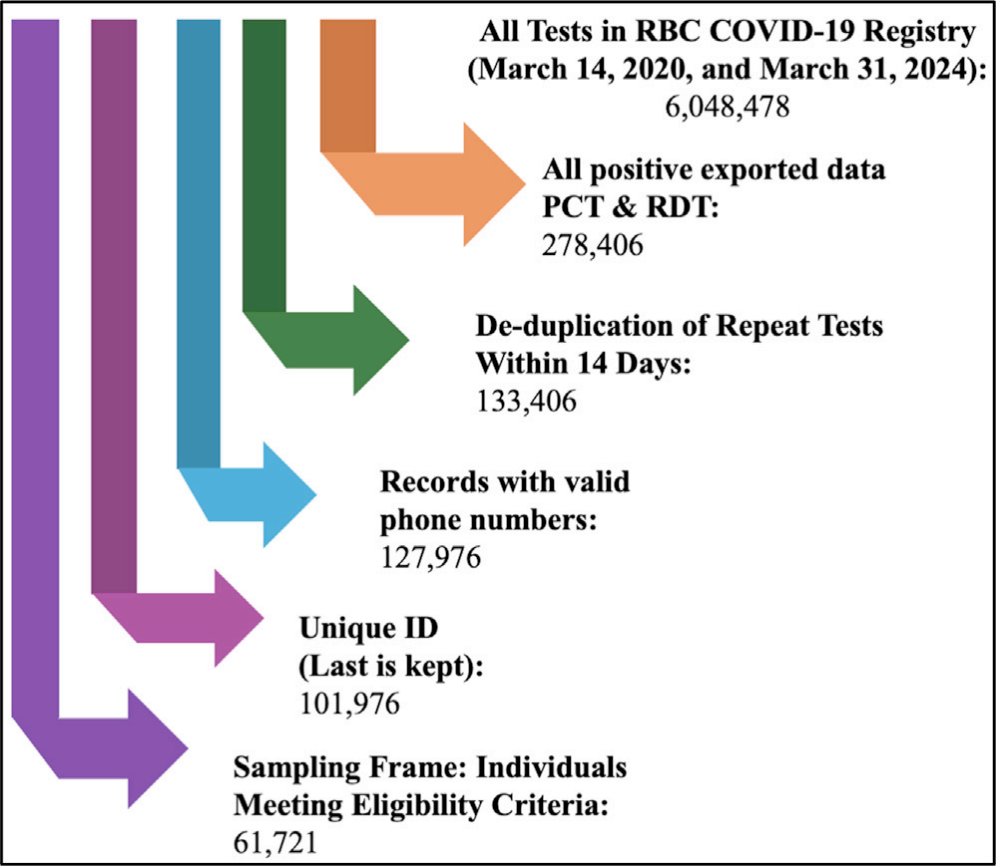
Flow diagram of participant selection from the RBC COVID-19 testing registry (March 14, 2020-March 31, 2024). PCT, Polymerase Chain Reaction Test; RBC, Rwanda Biomedical Centre; RDT, Rapid Diagnostic Test.

After applying all eligibility criteria, the final sampling frame consisted of 61,721 individuals, as detailed in Figure 1.

### Variables

PASC+, our dependent variable, is defined as being present if the recruited COVID-19 survivor:

• Reported at least one of the symptoms listed as being present at the time of the interview
• Experienced that symptom for at least 2 months
• Reported symptom onset within 3 months after their last positive COVID-19 test
• Was not experiencing the symptom prior to their COVID-19 infection.

Participants in this study are classified as either PASC+ or PASC– to assess the prevalence of PASC, identify risk factors for it, characterize the condition, and describe the impacts of PASC+ on the daily lives of COVID-19 survivors.

### Data sources

The available system data from the RBC COVID-19 test registry was the initial source used to identify and provide contact information of individuals who have tested positive for COVID-19 and selected during the sampling process.

The primary data collection instrument was a structured questionnaire developed by adapting items from two established sources: the US CDC–funded *PReventing Emerging Infections through Vaccine EffectiveNess Testing (PREVENT) Project* and the International Severe Acute Respiratory and Emerging Infection Consortium (*ISARIC*) COVID-19 follow-up case report form. This approach ensured inclusion of internationally recognized PASC domains while allowing adaptation to the Rwandan context. The draft tool was reviewed by clinicians and epidemiologists from the Rwanda Biomedical Centre, the University of Rwanda–African Centre of Excellence in Data Science, and Thomas Jefferson University to ensure cultural and clinical relevance.

Survey domains included demographic and background information, comorbidities, symptoms, and health care utilization. A large list of potential PASC symptoms, commonly reported in other cohorts, was presented, and respondents were requested to confirm if they experienced these or not and describe the temporality and duration if experienced. Participants completed the survey through a phone call with trained data collectors. The survey was conducted in Kinyarwanda, the dominant language in the country, to ensure clarity and understanding.

The tool was developed in English, translated into Kinyarwanda by professional translators, and back-translated to ensure conceptual accuracy. It was pilot-tested with 109 individuals from the national registry to refine question wording, skip patterns, and ensure appropriate language. Final revisions incorporated feedback to improve clarity and flow.

All interviews were conducted by trained data collectors via telephone in Kinyarwanda, with responses recorded directly into a secure REDCap database [7]. Variables extracted from the registry (test date, age, sex, province, reason for testing) were considered clinically validated. All other information, including comorbidities, symptoms, health care utilization, vaccination history, and functional impacts, was self-reported by participants.

### Sampling technique

The sample size was calculated using the Scalex SP calculator (Naing et al., 2022) [8], assuming a 20% prevalence of PASC based on the WHO estimates (2021), with a 2% margin of error and a 95% confidence interval (CI). To account for potential non-response (23%) and variability in prevalence over time, a design effect of 1.5 was applied, resulting in a final target sample size of 3,117 participants. A simple random sample was then drawn from this frame to meet the study’s calculated sample size requirements.

### Quantitative variables and statistical methods

Descriptive statistics were used to summarize participant characteristics and the prevalence of PASC in the sample, reported as a proportion with a 95% CI. Bivariate analysis (chi-square) identified potential associations between PASC and demographic, clinical, and COVID-19-related factors, further refined through a multivariate logistic regression and stepwise model reduction process to define the adjusted odds ratios (aOR) and 95% CI for each associated independent variable. A *P*-value of <0.05 was considered statistically significant. Data were analyzed using R Version 4.1.0 [9].

## Results

### Participants

Of the 3394 individuals contacted from the eligible sampling frame, 81 declined to participate, and 170 provided incomplete information. A total of 3143 participants completed the survey, yielding a response rate of 92.6%. This met and marginally surpassed the prespecified minimum sample size of 3,117, ensuring adequate statistical power.

### Descriptive data

Among the participants, 1265 (40%) were male and 1878 (60%) were female. By age group, 956 (30%) were between 18 and 34 years, 1312 (42%) were aged 35-49 years, and 874 (28%) were 50 years or older.

Date of the index COVID-19 infection was used as a surrogate to represent the most likely variant of the virus with respondents infected between March 14, 2020 and June 30, 2021 as one category (pre-Delta), likely Delta variant infections from July 1, 2021 to November 30, 2021 until the Omicron variant appears in Rwanda as of December 15, 2021 and persisted until the latest infection in our cohort on 09 January 2024.

Delta wave (July-November 2021) showed the highest proportion of PASC cases: 38.9% of infections led to PASC compared with 23.9% in the pre-Delta period (March 2020-June 2021) and 29.5% during Omicron (December 2021).

All of the respondents who were PASC+ continued to have at least one symptom on the date of enrollment in this study; thus, we analyzed the number of days that had elapsed between the index COVID-19 test and their enrollment/interview. Elapsed time to interview was similar between participants PASC+ and PASC- participants (median [interquartile range]: 1098 [1009-1138] vs 1106 [1010-1134] days); the only notable difference was an earlier minimum in the PASC+ group (252 vs 604 days).

**Figure 2:**
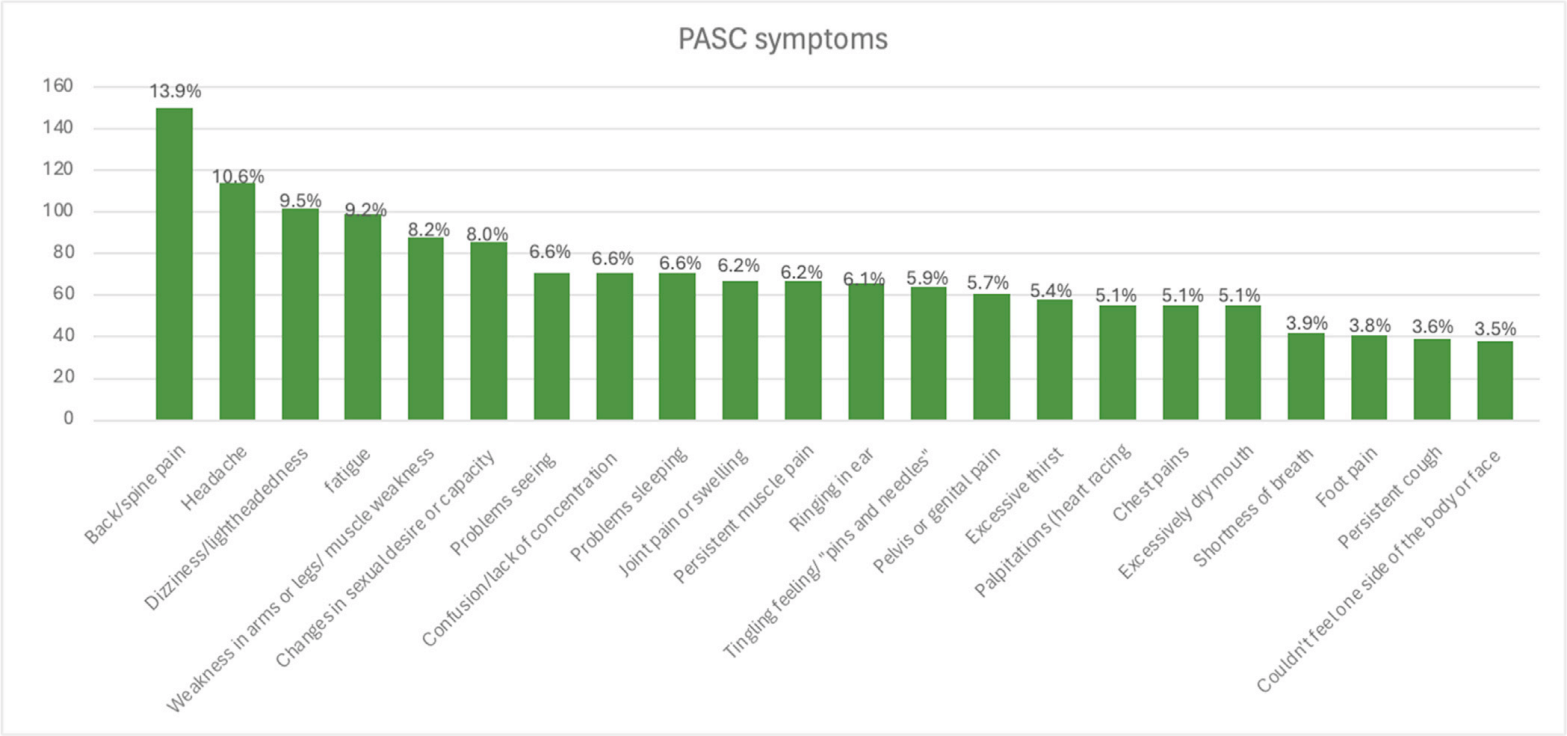
Frequency (%) of key PASC symptoms among COVID-19 survivors in Rwanda. PASC, post-acute sequelae of SARS-CoV-2 infection.

Of the 1076 PASC+ individuals, 1067 (99.2%) reported three or more PASC symptoms. The most commonly reported symptoms were back pain 433 (13.8%), headache 318 (10.1%), dizziness or lightheadedness 297 (9.4%), fatigue 287 (9.1%), changes in sexual desire or capacity 246 (7.8%), muscle weakness in the arms or legs 239 (7.6%), vision problems 238 (7.5%), and confusion or lack of concentration 228 (7.3%), as shown in Figure 2.

Fewer than one in 10 participants with PASC reported no effect on work, family, or social life. Mild-to-moderate limitations were most common, affecting roughly one-quarter to one-third of respondents in each domain, with family and social activities slightly more affected than work. About one-fifth experienced marked or extreme disruption, particularly in work and social interactions. Thus, PASC interferes with multiple facets of daily living, and the severity of impact varies across domains.

### Outcome data

Among the 3143 participants, 1076 experienced PASC, resulting in an overall prevalence of 34%. In the bivariate analysis, several sociodemographic and clinical factors were significantly associated with PASC. These included female sex, older age, lower education levels, being divorced or widowed, farming or part-time work, alcohol consumption, hospitalization during acute COVID-19, having more than one COVID-19 episode, and infection during the Delta wave. Individuals with at least one chronic condition were also more likely to report PASC. In contrast, COVID-19 vaccination status and the number of doses received were not significantly associated (Table 1).

**Table 1:** Distribution of PASC and bivariate analysis of associated factors.

Variables	Individuals, N = 3143 (100%)		
	PASC- 2,067 (65.8%)	PASC+ 1,076 (34.2%)	*P*-value
**Sex**			
***Male****– N = 1265 (40%)*	880 (69.6%)	385 (30.4%)	**<0.001**
***Female****– N = 1878 (60%)*	1187 (63.2%)	691 (36.8%)	
**Province**			
*Kigali City N =* 382 (12.1%)	286 (74.9%)	96 (25.1%)	**<0.001**
*Eastern N =* 855 (27.2%)	495 (57.9%)	360 (42.1%)	
*Northern N =* 720 (22.9%)	510 (70.8%)	210 (29.2%)	
*Southern N =* 573 (18.2%)	356 (62.1%)	217 (37.9%)	
Western N = 613 (19.5%)	420 (68.4%)	193 (31.6%)	
**Age category**			
*18-34 years N =* 956 (30%**)**	701 (73.3%)	255 (26.7%)	**<0.001**
*35-49 years N =* 1312 (42%)	844 (64.3%)	468 (35.7%)	
*50+ years N =* 874 (28%)	521 (59.6%)	353 (40.4%)	
**Education level**			
*None N = 380 (12%)*	209 (55.0%)	171 (45.0%)	**<0.001**
*Primary N = 1243 (40%)*	746 (60.0%)	497 (40.0%)	
*Secondary N = 643 (20%)*	455 (70.8%)	188 (29.2%)	
*University N = 709 (23%)*	554 (78.1%)	155 (21.9%)	
*Vocational training only N = 107 (3%)*	68 (63.6%)	39 (36.4%)	
*Literacy classes only N = 61 (2%)*	35 (57.4%)	26 (42.6%)	
**Pre-COVID 19 occupation**			
*Working full-time N =* 1399 *(*45%*)*	1009 (72.1%)	390 (27.9%)	**<0.001**
*Working part-time N =* 129 *(*4%*)*	76 (58.9%)	53 (41.1%)	
*Farmer N =* 1211 *(*39%*)*	691 (57.1%)	520 (42.9%)	
*Student N =* 132 *(*4%*)*	106 (80.3%)	26 (19.7%)	
*Unemployed N =* 272 *(*9%*)*	185 (68.0%)	87 (32.0%)	
**Post-COVID 19 occupation**			
*Same as before N = 2635 (84%)*	1735 (65.8%)	900 (34.2%)	0.9337
*Different from before N = 507(16%)*	332 (65.5%)	175 (34.5%)	
**Marital status**			
*Single N =* 515 *(*16%*)*	385 (74.8%)	130 (25.2%)	**<0.001**
*Married N =* 2344 *(*75%*)*	1515 (64.6%)	829 (35.4%)	
*Divorced N =* 88 *(*3%*)*	49 (55.7%)	39 (44.3%)	
*Widowed N =* 195 *(*6%*)*	117 (60.0%)	78 (40.0%)	
**Smoking status**			
*Yes N =* 257 *(*8%*)*	157 (61.1%)	100 (38.9%)	0.1124
*No N =* 2885 *(*92%*)*	1910 (66.2%)	975 (33.8%)	
**Alcohol consumption**			
*Yes N =* 1030 *(*33%*)*	720 (69.9%)	310 (30.1%)	**<0.001**
*No N =* 2113 *(*67%*)*	1347 (63.7%)	766 (36.3%)	
**Body mass index category**			
*Underweight N =* 57 *(*2%*)*	28 (49.1%)	29 (50.9%)	**0.003**
*Normal weight N =* 796 *(*25%*)*	541 (68.0%)	255 (32.0%)	
*Overweight N =* 417 *(*13%*)*	303 (72.7%)	114 (27.3%)	
*Obese N =* 186 *(*6%*)*	132 (71.0%)	54 (29.0%)	
**At least one comorbidity**			
*Yes N =* 3061 *(*97.4%*)*	2023 (66.1%)	1038 (33.9%)	**<0.001**
*No N =* 82 *(*2.6%*)*	44 (53.7%)	38 (46.3%)	
**Hospitalized for COVID-19**			
*No N =* 2941 *(*93.6%*)*	1965 (66.8%)	976 (33.2%)	**<0.001**
*Yes N =* 201 *(*6.4%*)*	101 (50.2%)	100 (49.8%)	
**Vaccinated against COVID-19**			
*No N =* 16 *(*0.5%*)*	10 (62.5%)	6 (37.5%)	0.991
*Yes N =* 3127 *(*99.5%*)*	2057 (65.8%)	1070 (34.2%)	
**Number of vaccine doses**			
*1-2 doses N =* 299 *(*9.5%*)*	198 (66.2%)	101 (33.8%)	0.404
*3 doses N =* 2494 *(*79.4%*)*	1650 (66.2%)	844 (33.8%)	
*>3 doses N =* 333 *(*10.6%*)*	208 (62.5%)	125 (37.5%)	
**Number of COVID-19 episodes**			
*1 N =* 2988 *(*95.1%*)*	1981 (66.3%)	1007 (33.7%)	**0.006**
*>1 N =* 154 *(*4.9%*)*	85 (55.2%)	69 (44.8%)	
**COVID-19 Period**			
Pre-Delta (March 2020-June 2021) N = 322 (10.2%)	245 (76.1%)	77 (23.9%)	**<0.001**
Delta (July-November 2021) N = 1789 (56.9%)	1094 (61.1%)	695 (38.9%)	
Omicron (≥December 2021) N = 1032 (32.8%)	728 (70.5%)	304 (29.5%)	

PASC, post-acute sequelae of SARS-CoV-2 infection.

At least one comorbid chronic illness was reported by 3061 (97.4%) of participants. Table 2 shows that prior to adjusting for confounders several conditions were significantly associated with increased likelihood of PASC, including hypertension (38.4% vs 32.4%, *P* = 0.0011), HIV (36.8% vs 33.1%, *P* = 0.0457), chronic kidney disease (40.8% vs 33.2%, *P* = 0.0026), rheumatoid arthritis (45.8% vs 33.5%, *P* = 0.0009), depression (51.5% vs 33.9%, *P* = 0.0028), and anxiety disorders (59.0% vs 33.9%, *P* = 0.0011). Other conditions, such as cancer, asthma, stroke, and epilepsy, did not show statistically significant associations with PASC (Table 2).

**Table 2:** Pre-existing medical conditions and their association with PASC.

Medical condition	Individuals, N = 3143 (100%)		
	PASC = No (2067)	PASC = Yes (1076)	*P*-value
Hypertension			
*No N =* 2168 *(*69.0%*)*	1466 (67.6%)	702 (32.4%)	**0.0011**
*Yes N =* 975 *(31%)*	601 (61.6%)	374 (38.4%)	
HIV			
*No N =* 2212 *(*70.4%*)*	1479 (66.9%)	733 (33.1%)	**0.0457**
*Yes N =* 931 *(*29.6%*)*	588 (63.2%)	343 (36.8%)	
Chronic liver disease			
*No N =* 2656 *(*84.5%*)*	1764 (66.4%)	892 (33.6%)	0.0727
*Yes N =* 487 *(*15.5%*)*	303 (62.2%)	184 (37.8%)	
Diabetes mellitus (I or II)			
*No N =* 2679 *(*85.2%*)*	1776 (66.3%)	903 (33.7%)	0.1337
*Yes N =* 464 *(*14.8%*)*	291 (62.7%)	173 (37.3%)	
Chronic kidney disease			
*No N =* 2734 *(*87.0%*)*	1825 (66.8%)	909 (33.2%)	**0.0026**
*Yes N =* 409 *(*13.0%*)*	242 (59.2%)	167 (40.8%)	
Cancer			
*No N =* 2932 *(*93.3%*)*	1934 (66.0%)	998 (34.0%)	0.3865
*Yes N =* 211 *(*6.7%*)*	133 (63.0%)	78 (37.0%)	
Allergic rhinitis			
*No N =* 2935 *(*93.4%*)*	1933 (65.9%)	1002 (34.1%)	0.6729
*Yes N =* 208 *(*6.6%*)*	134 (64.4%)	74 (35.6%)	
Rheumatoid arthritis			
*No N =* 2966 *(*94.4%*)*	1971 (66.5%)	995 (33.5%)	**0.0009**
*Yes N =* 177 *(*5.6%*)*	96 (54.2%)	81 (45.8%)	
Asthma			
*No N =* 2986 *(*95.0%*)*	1962 (65.7%)	1024 (34.3%)	0.7628
*Yes N =* 157 *(*5.0%*)*	105 (66.9%)	52 (33.1%)	
Heart failure			
*No N =*2 992 *(*95.2%*)*	1978 (66.1%)	1014 (33.9%)	0.0701
*Yes N =* 151 *(*4.8%*)*	89 (58.9%)	62 (41.1%)	
Chronic obstructive pulmonary disease			
*No N =* 3014 *(*95.9%*)*	1979 (65.7%)	1035 (34.3%)	0.5490
*Yes N =* 129 *(*4.1%*)*	88 (68.2%)	41 (31.8%)	
Depression			
*No N =* 3077 *(*97.9%*)*	2035 (66.1%)	1042 (33.9%)	**0.0028**
*Yes N =* 66 *(*2.1%*)*	32 (48.5%)	34 (51.5%)	
Anxiety disorders			
*No N =* 3104 *(*98.8%*)*	2051 (66.1%)	1053 (33.9%)	**0.0011**
*Yes N =* 39 *(*1.2%*)*	16 (41.0%)	23 (59.0%)	
Stroke			
*No N =* 3115 *(*99.1%*)*	2050 (65.8%)	1065 (34.2%)	0.5715
*Yes N =* 28 *(*0.9%*)*	17 (60.7%)	11 (39.3%)	
Epilepsy			
*No N =* 3122 *(*99.3%*)*	2052 (65.7%)	1070 (34.3%)	0.5832
*Yes N =* 21 *(*0.7%*)*	15 (71.4%)	6 (28.6%)	
Post-traumatic stress disorder			
*No N =* 3127 *(*99.5%*)*	2060 (65.9%)	1067 (34.1%)	0.0630
*Yes N =* 16 *(*0.5%*)*	7 (43.8%)	9 (56.3%)	

PASC, post-acute sequelae of SARS-CoV-2 infection.

All significant variables from the bivariate analysis were entered into a multivariate logistic regression model.

### Main results

**Figure 3:**
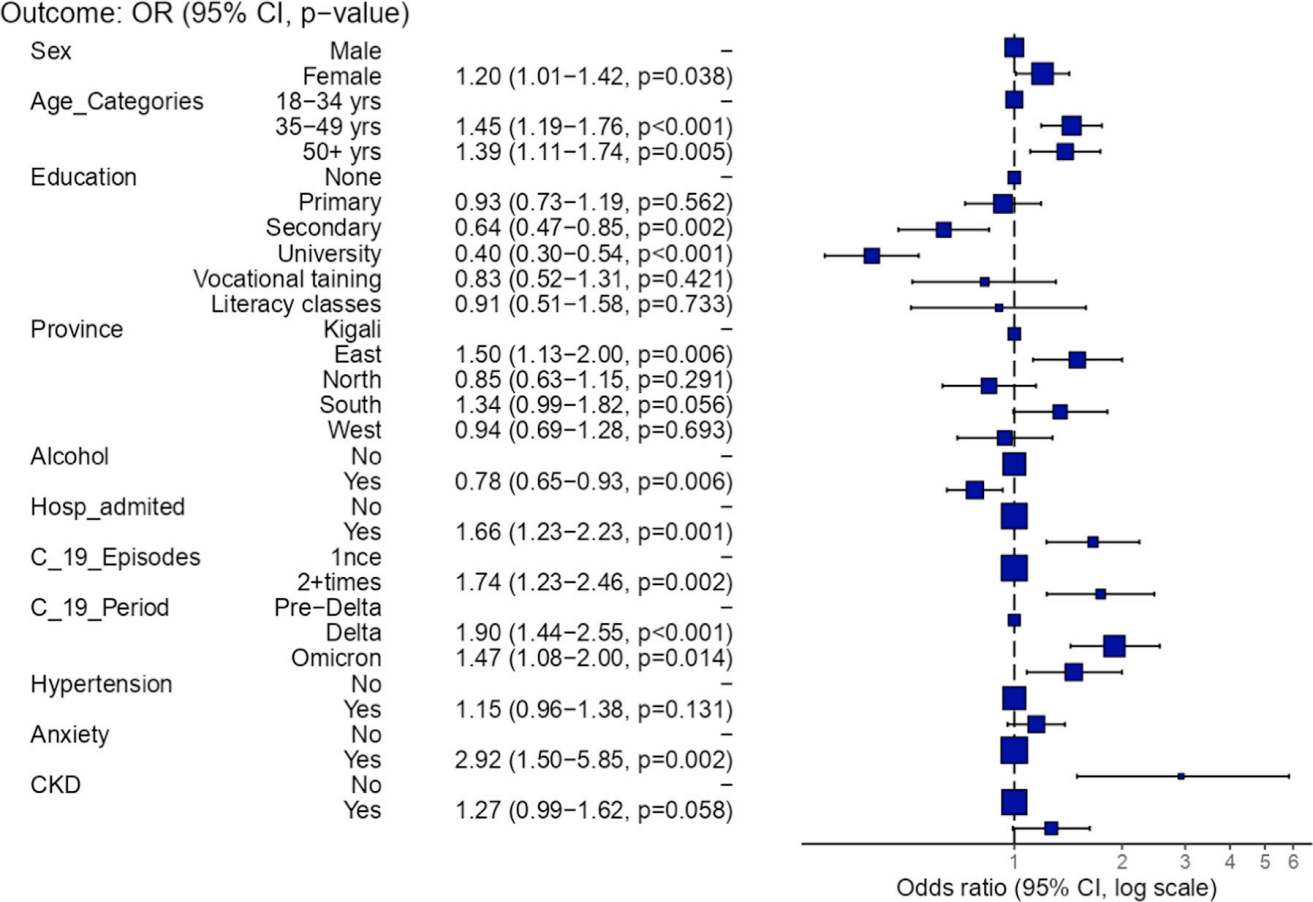
Multivariable logistic regression model of factors associated with PASC. CI, confidence interval; CKD, chronic kidney disease; OR, odds ratio; PASC, post-acute sequelae of SARS-CoV-2 infection.

In the final reduced model, female sex, older age groups (35-49 and 50+ years), lower education levels, residing in the Eastern Province residence, occupation, hospital admission during acute COVID-19, having multiple COVID-19 episodes, infection during the Delta or Omicron periods, and having anxiety remained significantly associated with PASC. Anxiety emerged as the strongest predictor, with nearly a threefold increase in odds of PASC (adjusted OR [aOR] 2.92; 95% CI 1.50-5.84; *P* = 0.002). In contrast, alcohol consumption was associated with lower odds (aOR 0.78; 95% CI 0.65-0.93). No significant association was observed between PASC and vaccination status or number of vaccine doses received (Figure 3).

## Discussion

This nationally representative survey provides the first population-level estimate of PASC in Rwanda. Drawing a random sample of 3143 adults from the country’s 133,406 confirmed cases, we found that one in three survivors (34%) met the WHO case definition for PASC, and almost all affected individuals reported multiple ongoing symptoms that interfered with daily functioning. Musculoskeletal pain, headache, dizziness, fatigue, visual, and cognitive difficulties predominated. PASC risk clustered in women, adults ≥35 years, residents of the Eastern Province, and those with repeat infections or concurrent anxiety, infections during the Delta and Omicron periods, whereas current alcohol use was linked to lower odds. By leveraging Rwanda’s integrated national surveillance and digital health infrastructure, our study captures the full spectrum of infection severity and supports growing evidence of a substantial, heterogeneous PASC burden across the continent.

The prevalence of PASC in our cohort was 34%, with back pain, headache, dizziness/lightheadedness, fatigue, reduced sexual desire or capacity, limb muscle weakness, vision problems, and cognitive difficulties being the most frequently reported symptoms. Comparable African data span a similarly high range: a rapid review of 14 studies pooled a prevalence of 41% (26-56%) with fatigue, dyspnea, and cognitive issues dominant [10]; a meta-analysis of 16 studies from Ethiopia, Ghana, South Africa, and Zambia yielded 42.1% (29.9-55.4%) and highlighted pulmonary, neurocognitive, and sleep-related complaints [11]; a scoping review of 17 studies across nine countries placed most estimates between 31% and 40%, again emphasizing fatigue, dyspnea, brain fog, headache, and insomnia [12]. Together, these syntheses confirm a substantial yet heterogeneous PASC burden across the continent and underscore the need for harmonized, population-based research.

An increased risk of PASC was observed among females and older adults (age 35 and older), aligning with global evidence that demographic characteristics influence vulnerability to long-term symptoms. Previous studies have consistently reported that women are more likely to experience persistent post-COVID-19 symptoms, potentially due to differences in immune response and hormonal regulation [13,14]. Similarly, advancing age has been associated with impaired viral clearance and prolonged inflammation, which may contribute to chronic symptomatology [14,15]. The present findings reinforce the need for targeted screening and post-infection support strategies that account for demographic risk profiles.

Anxiety emerged as the strongest independent predictor of PASC in this nationally representative cohort, with individuals with anxiety having nearly threefold higher odds of reporting persistent post-COVID-19 symptoms compared with those without. This finding is consistent with previous large-scale international studies that identified anxiety as a key risk factor for PASC [16,17]. Chronic anxiety may contribute to immune dysregulation or heightened symptom perception, thereby prolonging recovery [18]. Integrating psychological assessment and support into PASC care models may therefore be essential.

In our study, infection during both the Delta and Omicron variant waves was significantly associated with increased odds of developing PASC compared with infections in the pre-Delta period. The aOR was the highest for Delta (aOR 1.89; *P* < 0.001) compared with Omicron (aOR 1.46; *P* = 0.015). These findings align with population-based studies reporting that although Omicron infections are associated with a lower risk of PASC medical complaints compared with Delta, they still pose a higher burden than among uninfected individuals [19,20]. Although the overall prevalence of PASC appears broadly similar across major variants, specific Delta and Omicron sub-lineages have been linked to more persistent and severe symptom profiles [21]. A recent resurgence of COVID-19 in Rwanda, reflected in a 0.7% positivity rate among tested individuals, primarily linked to international travel, coincides with the global spread of the new NB.1.8.1 variant [22]. According to the WHO, despite increases in cases and hospitalizations in countries where NB.1.8.1 is widespread, there is currently no evidence that this variant causes more severe illness than other circulating variants [23]. These developments underscore the continued importance of variant-informed surveillance and long-term preparedness for the clinical and public health impacts of PASC.

Multiple studies have reported a protective effect of higher educational attainment against PASC [24,25] Our findings corroborate this gradient: individuals with high school and university education levels were less likely to report PASC compared with respondents with no educational attainment. Supporting this, evidence from global health literature has consistently shown that individuals with higher education are more likely to seek health care, engage with follow-up systems, and make informed decisions about their care [26]. These findings underscore the need for targeted post-COVID-19 support for socio-economically disadvantaged groups.

Our study did not find a significant association between COVID-19 vaccination and reduced prevalence of PASC. This result aligns with findings from a Dutch cohort study [27] and a Moroccan case-control study [28], both of which reported no therapeutic benefit of vaccination among individuals who had already experienced COVID-19. In contrast, several large population-based investigations [29,30] have demonstrated protective effects of vaccination when administered before infection, showing lower odds or prevalence of PASC among vaccinated individuals. Because vaccination coverage in our cohort was very high (99%), closely matching the national rate, there was limited variation in vaccination status. This lack of variation reduces the ability to detect statistically significant differences between groups, particularly if any true effect of vaccination on PASC risk is small.

### Strengths and limitations

Key strengths reinforce the credibility and policy relevance of our findings. First, the study draws on Rwanda’s national COVID-19 testing registry, enabling true nationwide coverage across all five provinces and both urban and rural settings, one of the few population-level PASC datasets in Africa. Second, we achieved a large analytic sample (n = 3143), providing ample statistical power and narrow confidence intervals that stabilize prevalence estimates. Third, participants were selected directly from the national test database rather than from hospital clinics; thus, the cohort captures the full spectrum of SARS-CoV-2 infection severity, including non-hospitalized cases that dominate community transmission but are often overlooked.

PASC character and presence were self-reported; thus, some respondents may have misattributed long-standing or unrelated ailments to COVID-19; this risk is heightened by the long recall window, nearly 3 years on average between infection and interview. In addition, our telephone survey necessarily excluded individuals who lacked a reachable phone or who died before data collection, potentially omitting those with more severe disease and leading to an underestimate of the true PASC burden. Nevertheless, the study’s large, nationally representative sample of 3143 adults, drawn from every province, provides ample statistical power and narrow confidence intervals, helping to stabilize prevalence estimates and temper the influence of random error and unmeasured selection bias.

## Ethical considerations

Ethical approval (RNEC542/2024) for this study was obtained from the Rwanda National Ethics Committee prior to implementation. Informed consent was obtained from all participants.

## Funding

Funding for this study was provided by Pfizer Global Medical Grants through the 2022/2023 COVID-19 ASPIRE Competitive Grant Program (Grant ID 77606011). The funder provided financial support only, they had no role in study design, data collection, data analysis, data interpretation, or writing of the manuscript.

## CRediT authorship contribution statement

FR: Conceptualization, Funding acquisition, Study design, Methodology, Writing – original draft, Verified data reported in this article, project administration. **VUN:** Data curation, Writing – Review & editing. **GR:** Methodology, Formal analysis, Verified data reported in this article. **ErR:** Formal analysis. **EdR:** conceptualization. **LS:** Methodology, Writing – review & editing. **CR:** Formal analysis, Writing – review & editing. **EK:** Conceptualization, Funding acquisition, study design, Writing – original draft, project administration, Writing – reviewing and editing, Literature search.

## Declaration of competing interest

The authors have no competing interests or disclosures.

## Appendix Supplementary materials

Image, application 1

Image, application 2

Image, application 3

Image, application 4
