# Supplementary figures and images for: Prevalence and characterization of post-acute sequelae of SARS-CoV-2 infection (PASC) in Rwanda
Source: IJID Reg. 2025 Sep 24;17:100738. doi: 10.1016/j.ijregi.2025.100738 (PMC12506432; doi:10.1016/j.ijregi.2025.100738)

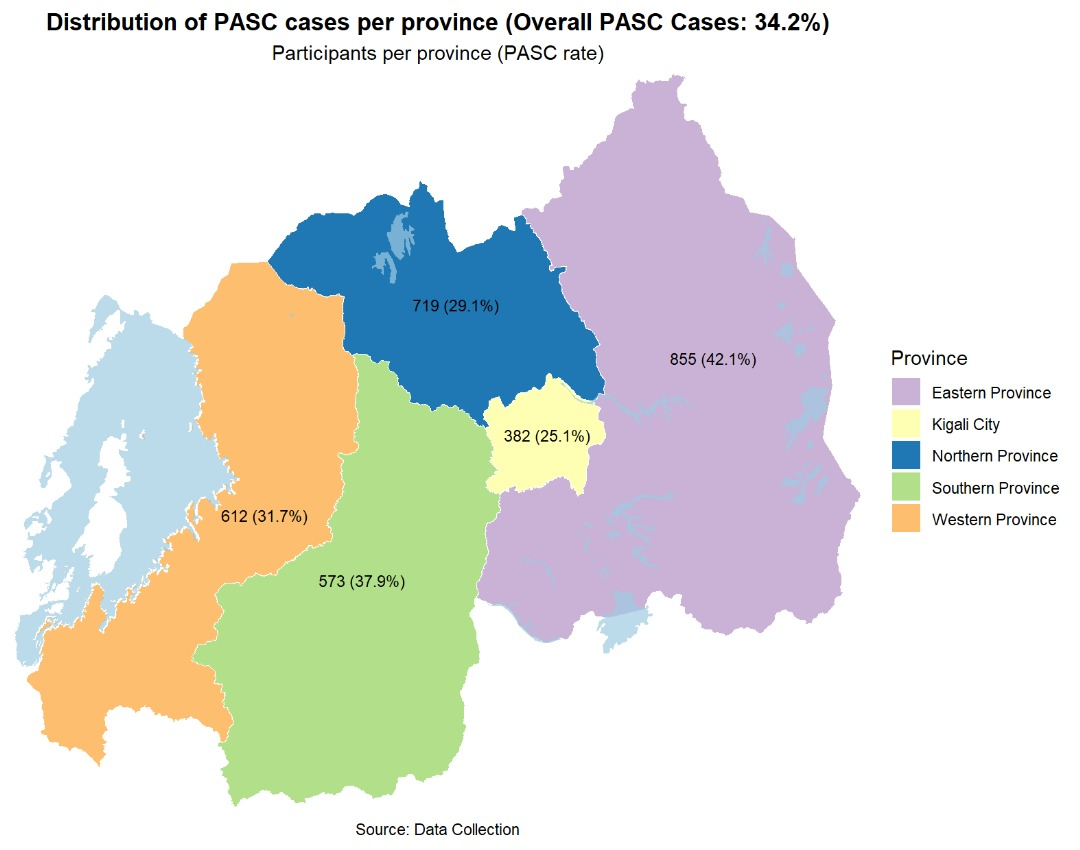
*Supplementary Figure S1: Distribution of PASC+ cases per province in Rwanda*

Supplement: Supplementary file 1 [file mmc1.docx]

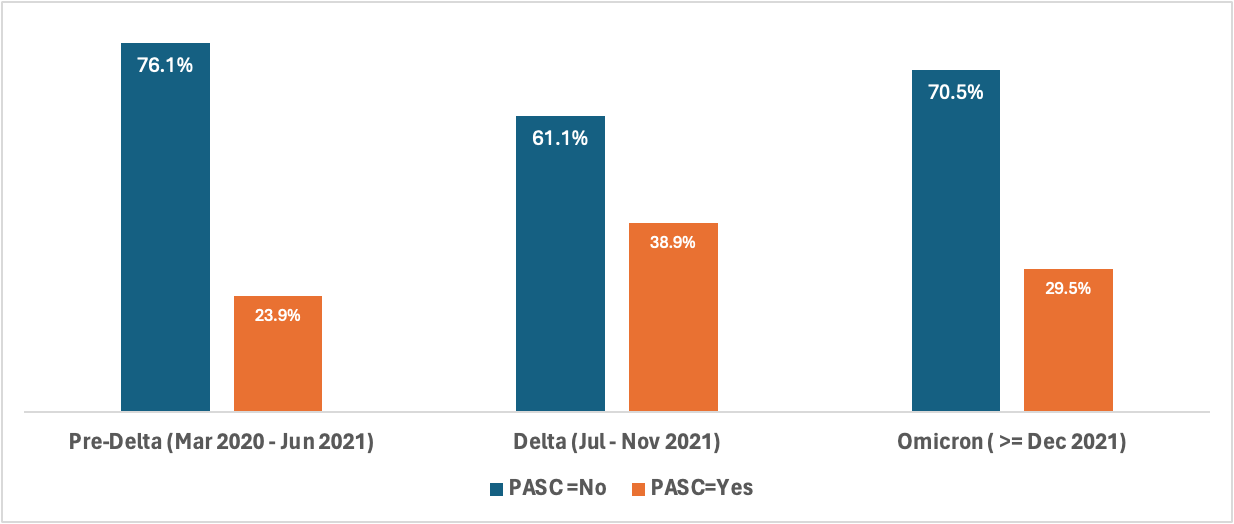
*Supplementary Figure S2. Temporality of COVID-19 cases in sample*

Supplement: Supplementary file 2 [file mmc2.docx]
