# Supplementary material for: Prevalence and characterization of post-acute sequelae of SARS-CoV-2 infection (PASC) in Rwanda
Source: IJID Reg. 2025 Sep 24;17:100738. doi: 10.1016/j.ijregi.2025.100738 (PMC12506432; doi:10.1016/j.ijregi.2025.100738)
Supplement: Supplementary file 3 [file mmc3.docx]

| **Supplementary Table S1. Days elapsed from initial SARS-CoV-2 diagnosis to interview, by PASC status** | | | | | | |
| --- | --- | --- | --- | --- | --- | --- |
| N = 3,143 | Minimum days | 1st Qu. | Mean days | Median days | 3rd Qu. | Max days |
| PASC - (2,067) | 604 | 1009 | 1081 | 1098 | 1138 | 1378 |
| PASC + (1,076) | 252 | 1010 | 1079 | 1106 | 1134 | 1348 |
