# Supplementary material for: Prevalence and characterization of post-acute sequelae of SARS-CoV-2 infection (PASC) in Rwanda
Source: IJID Reg. 2025 Sep 24;17:100738. doi: 10.1016/j.ijregi.2025.100738 (PMC12506432; doi:10.1016/j.ijregi.2025.100738)
Supplement: Supplementary file 4 [file mmc4.docx]

| **Supplementary Table S2. Multivariable logistic regression: full and reduced models of factors associated with PASC** | | | | | | | | |
| --- | --- | --- | --- | --- | --- | --- | --- | --- |
| **Variable** | **Full Model** | | | | **Reduced Model (Stepwise)** | | | |
|  | **Odd ratio** | **95% C.I** | | **P-value** | **Odd ratio** | **95% C.I** | | **P-value** |
| **Intercept** | 0.400 | 0.251 | 0.634 | 0.001 | 0.247 | 0.154 | 0.309 | **<0.001** |
| **Sex** |  | | | |  | | | |
| *Male (ref)* | 1 |  |  |  | 1 |  |  |  |
| *Female* | 1.192 | 0.997 | 1.426 | 0.054 | 1.204 | 1.014 | 1.429 | **0.034** |
| **Age category** |  | | | |  | | | |
| *18-34 Years (ref)* | 1 |  |  |  | 1 |  |  |  |
| *35-49 Years* | 1.343 | 1.088 | 1.662 | 0.006 | 1.441 | 1.186 | 1.753 | **<0.001** |
| *50+ Years* | 1.334 | 1.036 | 1.718 | 0.026 | 1.387 | 1.106 | 1.739 | **0.005** |
| **Education level** |  | | | |  | | | |
| *None (ref)* | 1 |  |  |  | 1 |  |  |  |
| *Primary* | 0.928 | 0.724 | 1.189 | 0.553 | 0.928 | 0.727 | 1.187 | 0.553 |
| *Secondary* | 0.704 | 0.514 | 0.963 | **0.028** | 0.636 | 0.475 | 0.851 | **0.002** |
| *University* | 0.461 | 0.329 | 0.646 | **<0.001** | 0.401 | 0.296 | 0.542 | **<0.001** |
| *Vocational training* | 0.873 | 0.541 | 1.394 | 0.573 | 0.828 | 0.519 | 1.308 | 0.423 |
| *Literacy classes* | 0.931 | 0.526 | 1.630 | 0.803 | 0.908 | 0.515 | 1.584 | 0.735 |
| **Occupation** |  | | | |  | | | |
| *Full-time (ref)* | 1 |  |  |  |  |  |  |  |
| *Part-time* | 1.473 | 0.991 | 2.177 | 0.053 |  |  |  |  |
| *Farmer* | 1.225 | 0.986 | 1.523 | 0.067 |  |  |  |  |
| *Student* | 0.848 | 0.515 | 1.360 | 0.961 |  |  |  |  |
| *Unemployed* | 0.992 | 0.729 | 1.343 | 0.961 |  |  |  |  |
| ***Location (Province)*** | | | | |  | | | |
| *Kigali (ref)* | 1 |  |  |  |  |  |  |  |
| *East* | 1.430 | 1.072 | 1.917 | **0.016** | 1.501 | 1.130 | 2.003 | **0.005** |
| *North* | 0.810 | 0.597 | 1.101 | **0.175** | 0.848 | 0.629 | 1.148 | 0.282 |
| *South* | 1.274 | 0.939 | 1.734 | **0.122** | 1.343 | 0.994 | 1.822 | 0.056 |
| *West* | 0.894 | 0.655 | 1.222 | **0.479** | 0.941 | 0.694 | 1.280 | 0.697 |
| **Alcohol consumption** | | | | |  | | | |
| *No (ref)* | 1 |  |  |  | 1 |  |  |  |
| *Yes* | 0.777 | 0.649 | 0.930 | **0.006** | 0.779 | 0.651 | 0.932 | **0.006** |
| **Admitted at hospital** | | | | |  | | | |
| *No (ref)* | 1 |  |  |  | 1 |  |  |  |
| *Yes* | 1.627 | 1.205 | 2.196 | **0.001** | 1.660 | 1.231 | 2.236 | **0.001** |
| **Number of C-19 Episodes** | | | | |  | | | |
| *Once (ref)* | 1 |  |  |  | 1 |  |  |  |
| *2+time* | 1.726 | 1.217 | 2.441 | **0.002** | 1.745 | 1.233 | 2.464 | **0.002** |
| **Covid-19 Period** |  |  |  |  |  |  |  |  |
| *Pre-Delta (Mar 2020 - Jun 2021) ref* | 1 |  |  |  |  |  |  |  |
| *Delta (Jul - Nov 2021)* | 1.629 | 1.206 | 2.198 | **0.001** | 1.897 | 1.430 | 2.542 | **<0.001** |
| *Omicron (>=Dec 2021)* | 1.727 | 1.218 | 2.443 | **0.002** | 1.462 | 1.081 | 1.994 | **0.015** |
| **Hypertension** |  | | | |  | | | |
| *No (ref)* | 1 |  |  |  | 1 |  |  |  |
| *Yes* | 1.133 | 0.915 | 1.403 | **0.251** | 1.154 | 0.960 | 1.387 | 0.127 |
| **Anxiety1** |  | | | |  | | | |
| *No (ref)* | 1 |  |  |  | 1 |  |  |  |
| *Yes* | 2.491 | 1.194 | 5.287 | **0.016** | 2.921 | 1.495 | 5.841 | **0.002** |
| **CKD1** |  | | | |  | | | |
| *No (ref)* | 1 |  |  |  | 1 |  |  |  |
| *Yes* | 1.236 | 0.955 | 1.600 | 0.107 | 1.270 | 0.992 | 1.625 | 0.057 |
| **HIV1** |  | | | |  | | | |
| *No (ref)* | 1 |  |  |  |  |  |  |  |
| *Yes* | 1.022 | 0.818 | 1.275 | 0.847 |  |  |  |  |
| **Rheumatoid1** |  | | | |  | | | |
| *No (ref)* | 1 |  |  |  |  |  |  |  |
| *Yes* | 1.177 | 0.843 | 1.639 | 0.336 |  |  |  |  |
| **Depression1** |  | | | |  | | | |
| *No (ref)* | 1 |  |  |  |  |  |  |  |
| *Yes* | 1.338 | 0.754 | 2.358 | 0.315 |  |  |  |  |
| **Marital status** |  | | | |  | | | |
| *Single (ref)* | 1 |  |  |  |  |  |  |  |
| *Married* | 1.143 | 0.887 | 1.479 | 0.305 |  |  |  |  |
| *Divorced* | 1.398 | 0.840 | 2.314 | 0.194 |  |  |  |  |
| *Widowed* | 0.896 | 0.588 | 1.365 | 0.611 |  |  |  |  |
